# Acute recoordination rather than functional hemodynamic improvement determines reverse remodelling by cardiac resynchronisation therapy

**DOI:** 10.1007/s10554-021-02174-7

**Published:** 2021-02-05

**Authors:** Philippe C. Wouters, Geert E. Leenders, Maarten J. Cramer, Mathias Meine, Frits W. Prinzen, Pieter A. Doevendans, Bart W. L. De Boeck

**Affiliations:** 1 University Medical Center Utrecht, Heidelberglaan 100, 3584 CX Utrecht, The Netherlands; 2 Maastricht University, P.O. Box 616, 6200 MD Maastricht, The Netherlands; 3 Luzerner Kantonsspital, 6000 Luzern, Switzerland

**Keywords:** Heart failure, Echocardiography, Dyssynchrony, dP/dt, CRT, Reverse remodelling

## Abstract

*Purpose*: Cardiac resynchronisation therapy (CRT) improves left ventricular (LV) function acutely, with further improvements and reverse remodelling during chronic CRT. The current study investigated the relation between acute improvement of LV systolic function, acute mechanical recoordination, and long-term reverse remodelling after CRT. *Methods*: In 35 patients, LV speckle tracking longitudinal strain, LV volumes & ejection fraction (LVEF) were assessed by echocardiography before, acutely within three days, and 6 months after CRT. A subgroup of 25 patients underwent invasive assessment of the maximal rate of LV pressure rise (dP/dt_max,_) during CRT-implantation. The acute change in dP/dt_max_, LVEF, systolic discoordination (internal stretch fraction [ISF] and LV systolic rebound stretch [SRSlv]) and systolic dyssynchrony (standard deviation of peak strain times [2DS-SD18]) was studied, and their association with long-term reverse remodelling were determined. *Results*: CRT induced acute and ongoing recoordination (ISF from 45 ± 18 to 27 ± 11 and 23 ± 12%, p < 0.001; SRS from 2.27 ± 1.33 to 0.74 ± 0.50 and 0.71 ± 0.43%, p < 0.001) and improved LV function (dP/dt_max_ 668 ± 185 vs. 817 ± 198 mmHg/s, p < 0.001; stroke volume 46 ± 15 vs. 54 ± 20 and 52 ± 16 ml; LVEF 19 ± 7 vs. 23 ± 8 and 27 ± 10%, p < 0.001). Acute recoordination related to reverse remodelling (r = 0.601 and r = 0.765 for ISF & SRSlv, respectively, p < 0.001). Acute functional improvements of LV systolic function however, neither related to reverse remodelling nor to the extent of acute recoordination. *Conclusion*: Long-term reverse remodelling after CRT is likely determined by (acute) recoordination rather than by acute hemodynamic improvements. Discoordination may therefore be a more important CRT-substrate that can be assessed and, acutely restored.

**Supplementary Information:**

The online version contains supplementary material available at 10.1007/s10554-021-02174-7.

## Introduction

Previous studies have demonstrated that the clinical benefits of cardiac resynchronization therapy (CRT) are accompanied by improvements of left ventricular (LV) function and reverse remodelling [1]. Based on the assumption that acute improvements in hemodynamic parameters both reflect effective resynchronisation and convey longer-term beneficial effects of CRT, acute increases in stroke volume and maximum rate of LV pressure rise (dP/dt_max_) have been applied to assess CRT-response since the very beginning of CRT development [2–6]. Invasive dP/dt_max_ has been used to quantify acute de- and resynchronisation effects in animal studies [7], to guide LV placement [8, 9], and to optimize atrioventricular and interventricular delay in the setting of clinical research [5, 10, 11]. On the contrary, measures of baseline mechanical dyssynchrony (i.e. temporal dispersion in myocardial shortening) and discoordination (i.e. reciprocal shortening and stretching) have been shown to identify the mechanical substrate of CRT and relate to longer-term benefits, but acute effects and response mechanisms have little been studied [12, 13]. Moreover, a direct comparison of these two approaches has not been performed yet.

A considerable part of the remodelling processes involved in CRT appears to be linked to local mechanics [14–16]. This comparative study therefore set out on the hypothesis that long-term improvement of LV function (i.e. ejection fraction) and reverse remodelling after CRT is determined by acute recoordination of LV contraction, rather than by a functional response characterised by acute hemodynamic improvements.

## Methods

### Study population and study protocol

A total of 35 patients with good echocardiographic image quality who underwent CRT implantation were prospectively included in the present study. All patients had severe symptomatic heart failure (New York Heart Association class [NYHA]) II–IV, LV ejection fraction (LVEF) < 35%) despite optimal medical therapy and had a QRS-width ≥ 120 ms with an LBBB-morphology. Transthoracic echocardiography was performed in each patient before, within 3 days after, and 6 months after CRT implantation. In a subgroup of 25 patients, device settings were optimized by maximizing the invasively determined maximal rate of LV pressure rise (dP/dt_max_) during the implantation procedure [17]. In the remaining 10 patients, algorithms based on the intracardiac electrogram implemented in the devices were used for device optimization [18]. Echocardiographic response to CRT was assessed by the reduction in LV end-systolic volume, with responders defined as patients with > 15% reduction at 6 months (“long-term”) follow-up [19].

### Echocardiographic and strain imaging protocol

Echocardiography was performed on a Vivid 7 ultrasound machine (General Electric, Milwaukee, USA) using a M3S transducer. A minimum of 3 loops were acquired at breath hold and analyzed offline (Echopac version 6.0.1, General Electric). Pulsed Doppler flow profiles at LV inflow (mitral valve opening & closure) and outflow (aortic valve opening & closure) as well as at the right ventricular outflow tract (pulmonary valve opening) were employed for cardiac event timing. Systole was defined as the interval between mitral and aortic valve closure. For deformation imaging, dedicated narrow sector single wall images of the septum, anteroseptum, anterior, lateral, posterior, and inferior wall were prospectively acquired at 51–109 frames per second. For each wall, longitudinal strain curves at the basal, midventricular and apical segments (i.e. 18-segment model) were derived by speckle tracking with the onset of the QRS as zero reference. The obtained traces were further post-processed along with cardiac event timing markers in a custom-made toolbox (STOUT: Speckle tracking Toolbox Utrecht), yielding spatially encoded and time-normalized deformation data over the entire LV [20]. Stroke volume (SV), LVEF, LV end-systolic (LVESV) and end-diastolic (LVEDV) volumes by biplane Simpson’s method were analysed by one (GL), and all deformation and dyssynchrony measurements independently by another (BDB) observer. Both were blinded to each other and to the invasive dP/dt_max_ measurements.

### Dyssynchrony and discoordination parameters

**Fig. 1:**
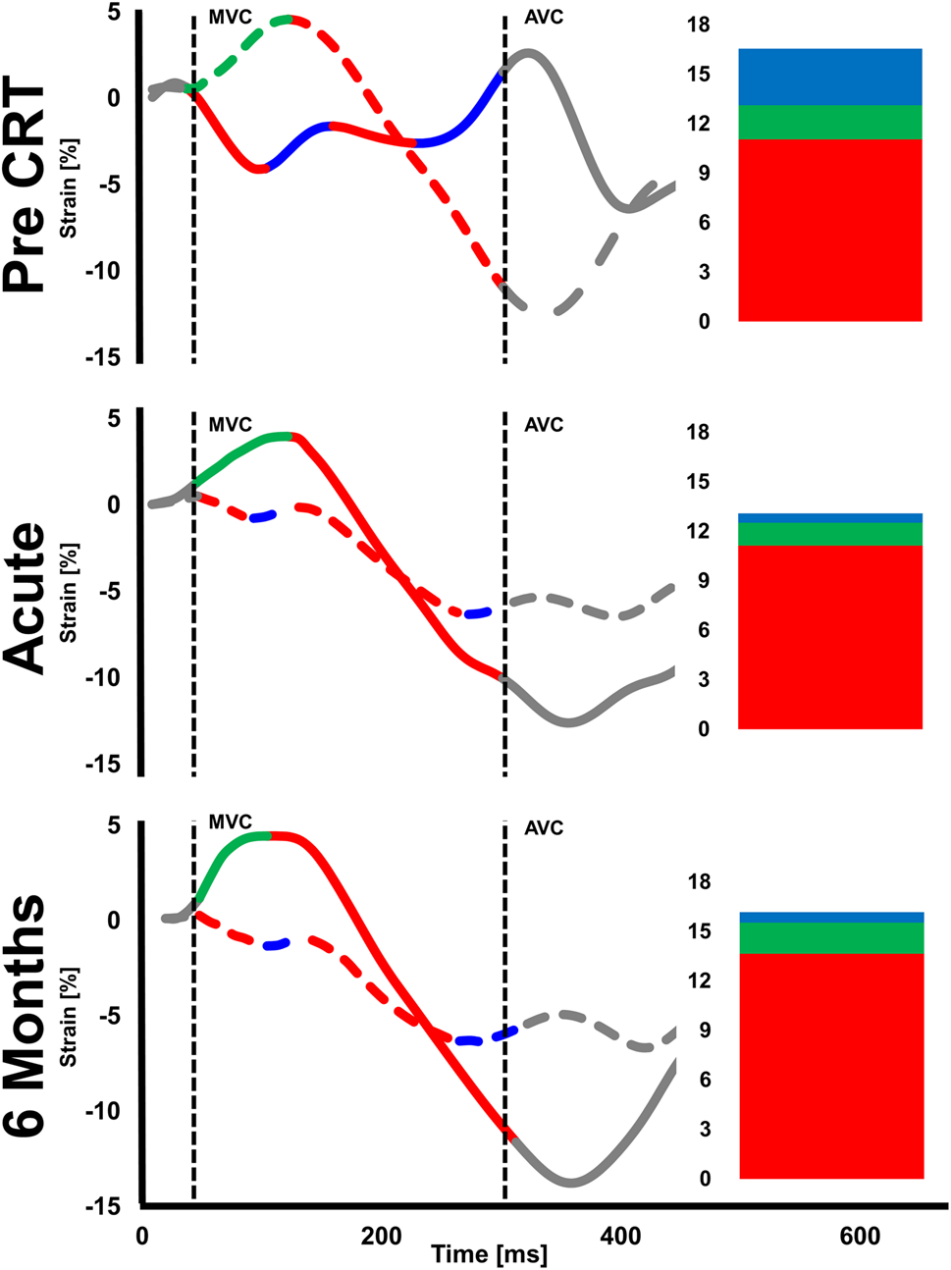
Systolic rebound stretch as part of a biphasic response in recoordination. Left ventricular systolic rebound stretch (SRSlv) is calculated as the total amount of systolic stretching that occurs after prematurely terminated shortening during systole (i.e. strain-amplitudes of positive longitudinal strain), averaged over the total number of segments. Compared to baseline, an acute reduction in the amount of systolic rebound stretch (blue; i.e. wasted work) of the left ventricle is seen upon biventricular pacing, without concomitant improvement in systolic shortening (red; i.e. constructive work). Conversely, improvement in systolic shortening becomes apparent after six months of prolonged biventricular stimulation. Note that for illustrative purposes only two segmental strain curves, both of the septum (solid lines) and lateral wall (dashed lines), are displayed for a representative CRT responder

Inter-ventricular mechanical delay (IVMD) was defined as the delay between pulmonary and aortic valve opening. Intra-ventricular LV-dyssynchrony was defined as the standard deviation of time to peak strain in all segments (2DS-SD18). As a surrogate marker of mechanical work-inefficiency, discoordination was assessed as the internal stretch fraction (ISF) and systolic rebound stretch (SRSlv) [21–23]. To calculate ISF, each strain rate curve was automatically split into their respective shortening (negative strain) and stretching (positive strain) components. The average strain rate of all LV segments was then determined for the these shortening and stretching components separately. The *fraction* of total systolic stretch (i.e. wasted work) relative to total systolic shortening (i.e. constructive work) determined ISF (Supplemental Fig. 1) [21]. Conversely, as a more specific index of systolic wasted work by itself, SRSlv was calculated. Here, all systolic stretch that occurs immediately after prematurely terminated shortening was summed and averaged over the total amount of LV segments (Fig. 1) [20, 23].

### LV dP/dt_max_ measurements during device implantation

Device implantation was performed under local anaesthesia. Right ventricular apical (RV) and atrial leads were placed transvenously at conventional positions. The LV lead was aimed at a tributary of the coronary sinus overlying the LV free wall. Leads were connected to a CRT-defibrillator in all patients.

LV dP/dt_max_ was obtained by placing a pressure wire (PressureWire™ Certus, St. Jude Medical Inc., St. Paul, MN, USA) in the LV via femoral arterial access. After baseline measurement of LV dP/dt_max_, the acute effect of CRT on LV dP/dt_max_ was derived automatically from continuous, digitized invasive pressure measurements digitized at 100 Hz (Radi Analyzer Physio Monitor v1.0 beta4, St. Jude Medical, Inc., St. Paul, MN, USA). Measurements were averaged over 10 s for each setting. Premature ventricular beats and the first post-extrasystolic beat were manually excluded from analysis. AV- and VV-delays were optimized to maximize the increase in LV dP/dt_max_ compared to baseline [17]. Paced AV-delays were shortened by 20 ms steps from 240 ms to 80 ms. VV-delay optimization was performed at the previously determined optimal AV-delay starting with LV pre-activation by 80 ms followed by lengthening of the VV delay by steps of 20 ms until RV pre-activation by 80 ms.

### Statistical analysis

Statistical analysis was performed using SPSS version 25.0 (SPSS Inc., Chicago, Illinois). Values are presented as mean and standard deviation (SD) for continuous variables, and as numbers and percentages for categorical variables. Assumptions on homogeneity of variances and normally distributed residuals were checked by Levene’s test and Q-Q plots respectively. Comparison of continuous data between responders and non-responders was performed by independent samples t-test. Comparison of continuous data over time was performed by repeated measurements analysis of variance (ANOVA). Categorical data were compared by chi-square or Fischer’s exact test as appropriate. Bonferroni post-hoc correction for multiple comparisons was applied when applicable. For the direct comparison of the relationship between acute improvements and long-term response, Pearson correlation were restricted to the subset of 25 patients with complete dP/dt_max_, dyssynchrony, and discoordination measurements. A p-value < 0.05 was considered statistically significant.

## Results

### Patient population

Mean age of the study population was 65 ± 11 years, 60% were male, 83% were in NYHA functional class III, heart failure aetiology was ischemic in 46%. LV lead placement was posterior or posterolateral in 15 (43%), lateral in 18 (51%) and anterolateral in 2 (6%) patients. Echocardiographic data including all volumetric, dyssynchrony and discoordination parameters at baseline, immediately after implant & at 6 months follow up was available for all patients (n = 35). The subgroup with LV dP/dt_max_-guided optimization comprised 25 patients enabling the direct comparison of acute LV dP/dt_max_ augmentation and acute recoordination with long-term response. Baseline LV dP/dt_max_ was determined during sinus rhythm (n = 20), atrial pacing (due to sick sinus, n = 2) and RV pacing (pacing-dependent AV-Block, n = 3) and was on average 668 ± 185 mmHg/s.

Baseline characteristics of this subgroup with dP/dt_max_ data as well as of the entire study population stratified according to response are provided in Supplemental Table 1. In total, 20 patients (57%) were classified as echocardiographic responder. Non-responders were similar to responders concerning all baseline clinical characteristics.

### Recoordination, hemodynamic improvement, and reverse remodelling after CRT

**Fig. 2:**
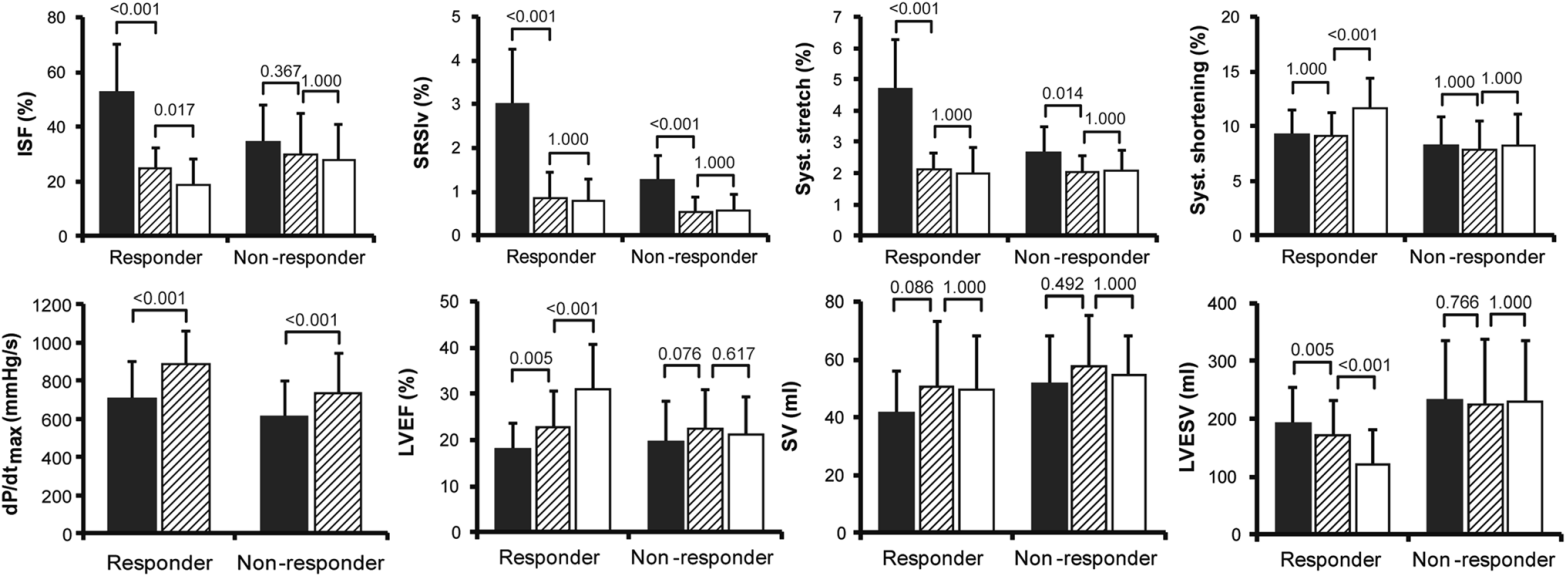
Evolution of discoordination and left ventricular hemodynamic function stratified according to response. Mean and standard deviation values of discoordination (upper panels) and parameters of left ventricular hemodynamic function and dimensions (lower panels). Results are shown before, directly after and six months after CRT (black, shaded, and white bars, respectively) in responders and non-responders. Note that acutely after CRT, coordination improves by reduction of systolic stretch, whereas during long-term follow-up systolic stretch remains stable but systolic shortening improves in responders. ISF, internal stretch fraction; SRSlv, systolic rebound stretch of the septum; dP/dt_max_, maximum rate of LV pressure rise; SV, stroke volume; LVEF, left ventricular ejection fraction; LVESV, LV end-systolic-volume

In the entire group, initiation of CRT acutely improved coordination (ISF & SRSlv) and synchrony (IVMD, 2DS-SD18) along with augmented LVEF, SV and LV dP/dtmax (Supplemental Table 2). Acute recoordination and resynchronisation were maintained over time. Interestingly, the acute improvement of LV coordination was exclusively caused by a reduction of paradoxical systolic stretch (both systolic stretch and SRSlv), at unchanged systolic shortening. Inversely, longer-term CRT further improved recoordination in terms of a further decline in ISF exclusively by an increase in the systolic shortening fraction without effect on paradoxical stretch. Stroke volume increased acutely upon starting CRT, but remained constant during 6 months follow-up, while LVEF continued to increase due to a balanced reduction in LVEDV and LVESV (Fig. 2 and Supplemental Fig. 2).

The effects of CRT on LV coordination, hemodynamic function and synchrony, stratified according to response, are displayed graphically in Fig. 2 and Supplemental Fig. 2. At baseline, responders had higher baseline values of discoordination (ISF 53 ± 17 vs. 35 ± 13%, p = 0.003; SRSlv 3.01 ± 1.26 vs. 1.28 ± 0.56%, p < 0.001) and dyssynchrony (IVMD 62 ± 20 vs. 39 ± 28 ms, p = 0.012; 2DS-SD18 158 ± 42 vs. 125 ± 43 ms, p = 0.026) compared to non-responders. As seen in the entire group, both responders and non-responders showed an immediate reduction in systolic stretch and IVMD upon CRT initiation, finally reaching similar values during acute CRT. However, in view of the lower baseline values, the absolute reduction in systolic stretch was smaller in non-responders and became insignificant when expressed in relative terms by ISF. Similarly, the reduction of 2D-SD18 by CRT did not reach significance in non-responders (Supplemental Fig. 2). Significant long-term improvement of myocardial shortening, with the associated further decrease in ISF, was only present in responders. Responders and non-responders showed a similar acute hemodynamic response, both expressed in terms of dP/dt_max_ and ejection fraction (Fig. 2). However, only in the responder group EF continued to rise during longer lasting CRT.

### Relation of acute improvements with long-term response

Acute recoordination parameters were closely related to reverse remodelling and ∆LVEF after 6 months (Table 1). The best predictor of reverse remodelling was the acute reduction of systolic rebound stretch within the LV (∆SRSlv; R = 0.767, p < 0.001). Similar relations with reverse remodelling were found for *baseline* measurements of discoordination (R = 0.796 and R = 0.581 for SRSlv and ISF, respectively: both p < 0.001) and dyssynchrony (R = 0.475, p = 0.005 and R = 0.518, p = 0.001 for IVMD and 2D-SD18 respectively). In contrast, acute increases in LV dP/dt_max_, LVEF and SV neither predicted long-term response (i.e. reverse remodelling and ∆LVEF) after 6 months (Table 1) nor did they correlate with the extent of acute recoordination and resynchronisation (Supplemental Table 3). Additionally, acute improvements in mitral regurgitation effective regurgitant orifice (∆MRero; R = 0.064, p = 0.778) and RV tricuspid annular plane systolic excursion (∆TAPSE; R = 0.101, p = 0.630) were unrelated to long-term reverse remodelling as well.

**Table 1:** Relation of acute improvements with reverse remodelling and changes in LVEF (n = 25)

Parameter	6-months ∆LVESV (%)		6-months ∆LVEF (%-point)	
	R	p-value	R	p-value
Acute recoordination				
∆ISF (%-point)	0.601	< 0.001	0.578	0.002
∆Systolic stretch (%-point)	0.676	< 0.001	0.628	< 0.001
∆SRSlv (%-point)	0.765	< 0.001	0.694	< 0.001
Acute resynchronisation				
∆IVMD (ms)	0.133	0.554	0.140	0.535
∆2D-SD18 (ms)	0.500	0.011	0.451	0.023
∆QRS (ms)	0.188	0.368	0.131	0.533
Acute improvement of systolic function				
∆dP/dt_max_ (%)	0.251	0.277	0.173	0.409
∆SV (%)	0.047	0.830	0.250	0.249
∆LVEF (%-point)	0.237	0.265	0.392	0.077

For a uniform representation, all changes ∆ express a physiologic improvement, i.e. decrements for discoordination & dyssynchrony parameters, and increments for function parameters such that relations are positive if both parameters improve

Abbreviations: *ISF* internal stretch fraction, *SRSlv* left ventricular systolic rebound stretch, *2DS-SD18* standard deviation of time to peak strain, *IVMD* interventricular mechanical delay, *LVEDV* left ventricular (*LV*) end-diastolic volume, *LVESV, LV* end-systolic volume, *SV* stroke volume, *LVEF, LV* ejection fraction, dP/dt_max_, maximum rate of LV pressure rise

## Discussion

**Fig. 3:**
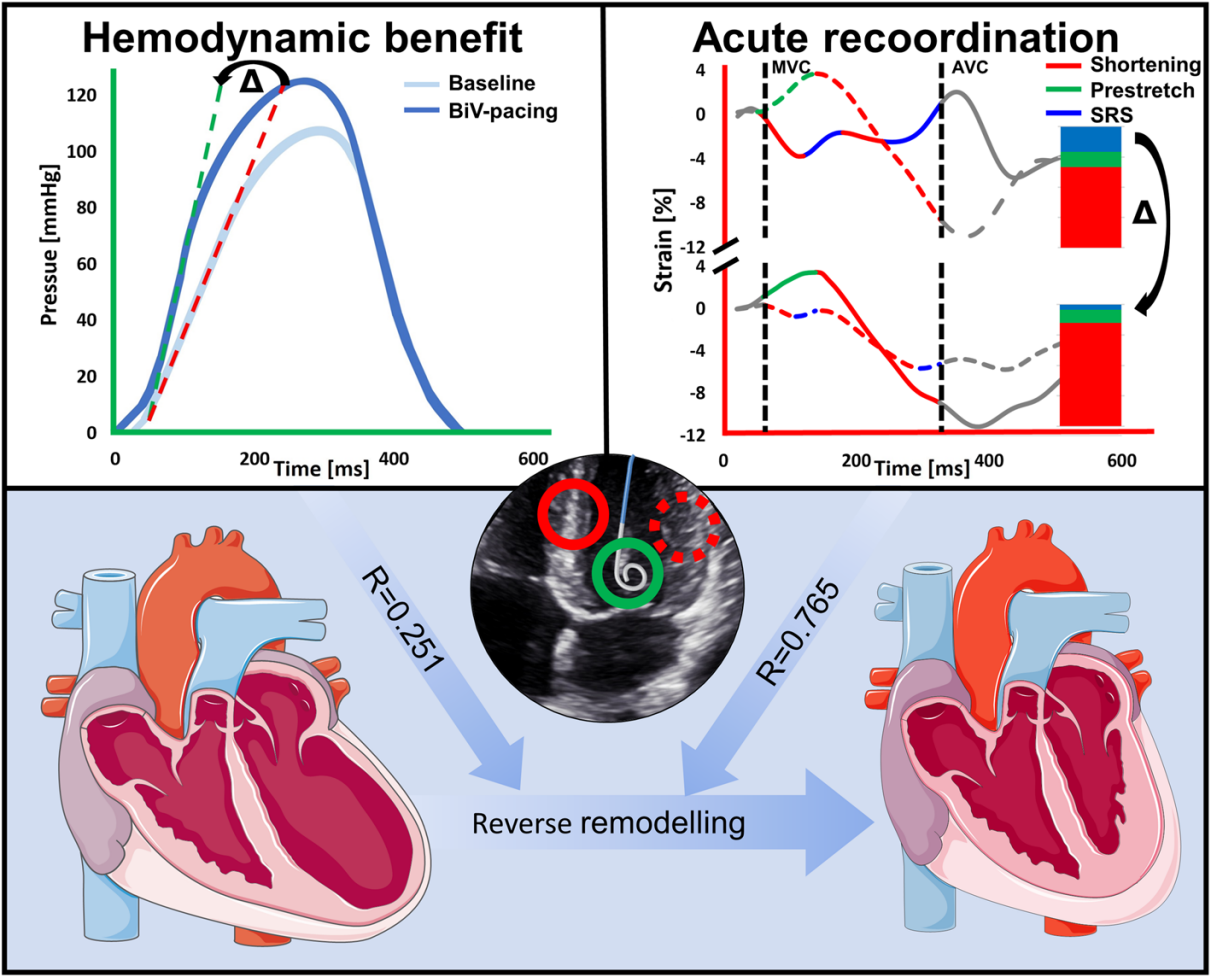
Visual abstract summarizing the main study methods and results. Biventricular (BiV) pacing elicited an acute LV functional response, reflected by an acute increase (∆) in invasively determined LV dP/dt_max_ (top left panel). BiV pacing also induced acute recoordination of LV deformation (top right panel), predominantly characterised by acute reduction (∆) in paradoxical systolic rebound stretch of the LV (SRSlv). As such, a smaller fraction of the work that was performed during systole was internally wasted by segments paradoxically stretching. This ensues in a lower internal strain fraction (ISF, not displayed), thereby signifying higher efficiency. When comparing acute improvements in LV systolic function and acute recoordination, the parameter most strongly related to reverse remodelling was the extent of acute recoordination, and not acute hemodynamic response

This study demonstrates that the extent of acute mechanical recoordination achieved upon CRT-initiation, specifically SRSlv, is highly predictive of volumetric response to CRT at 6 months follow-up. In contrast, our results substantiate that an acute functional response (i.e. hemodynamic improvement of LV systolic function) poorly relates to the extent of acute LV recoordination, and therefore poorly predicts longer-term remodelling (Fig. 3). Moreover, this study strongly suggests a bimodal response to CRT: an immediate alleviation of dyssynchrony and of paradoxical systolic stretch in particular, followed by a true improvement of myocardial shortening on the longer-term (Fig. 1). Taken together, these findings emphasize the importance of correction of local derangements in cardiac mechanics to trigger reverse remodelling after CRT.

### Acute functional hemodynamic improvements fail to predict long-term response

In the present study, acute hemodynamic changes, whether invasively assessed by LV dP/dt_max_, or echocardiographically by LVEF and stroke volume did not relate to resynchronisation and failed to predict long-term reverse remodelling. These data challenge the historical assumptions that the best achievable acute functional response, as reflected by e.g. dP/dt_max_, reflects optimal resynchronization, predicts a favorable longer-term outcome, and–in extenso–may represent targets for CRT-optimization. These assumptions predominantly followed numerous observations that CRT induces immediate and sustained improvements in both mechanical activation synchrony as well as in hemodynamic parameters, most notably dP/dt_max_ [2–6, 8, 9, 14, 24–27]. Nevertheless, preclinical evidence to support acute functional response as a predictor of longer-term remodelling is sparse and clinical patient data conflicting. Importantly, only one study suggested ∆dP/dt_max_ as a predictor of long-term reverse remodelling [28]. In contrast, Stellbrink et al. and Bogaard et al. independently demonstrated that volumetric and clinical responders to CRT, were found among those with both very high and with minimal acute ∆dP/dt_max_ [17, 29]. In analogy, echocardiographic subanalyses of the REVERSE-trial and the PROSPECT-trial demonstrated no relation between the acute increase in LVEF and long-term effects of CRT on reverse remodelling and clinical outcome [26, 30]. Therefore, acute and chronic improvements seem part of a heterogeneous spectrum of CRT response, subject to many additional variables. This notion is further supported by the absence of a clear relation between ∆dP/dt_max_ and acute recoordination in the present study.

### Acute reversal of paradoxical stretch and mechanical inefficiency mediate CRT response

The current study clearly suggests longer-term reverse remodelling to be significantly mediated by the extent to which CRT can acutely improve mechanical coordination through acute reversal of paradoxical systolic stretch. We have previously demonstrated that CRT response at 6 months involves a redistribution and homogeneization of systolic strain amplitudes by improving local and global myocardial shortening proportionally to the reduction in paradoxical systolic stretch [21]. The most specific stretch component associated with improved shortening and response in that study was the systolic rebound stretch that abunds at the septum [21]. To this end, the added prognostic value of high baseline SRS of the septum (and correction thereof), in addition to simple visual assessment of dyssynchrony, has recently been proven in 200 CRT recipients, both in patients with LBBB and non-LBBB [12].

Similar results have been obtained when evaluating changes in recoordination after 6 months in terms of strain-based myocardial work. Here, homogenization of systolic work was demonstrated primarily through mitigation of wasted (i.e. paradoxical) work [31]. Recently, Duchenne et al. demonstrated that acute redistribution of regional LV work, specifically from the lateral wall towards the septum, determined long-term reverse remodelling [32]. With regional systolic strain representing the main determinant of regional myocardial work in that study, redistribution of work through reversal of wasted work was based on reversal of septal paradoxical stretch also in that study. As opposed to the present study, indices of discoordination and/or myocardial work have not been directly compared to invasive hemodynamic measures such as dP/dt_max_ before. However, because both ISF and SRS of the septum at baseline show close correlation with the septum-to-lateral wall ratio of myocardial work, similar results are to be expected when using afterload-integrated indices of wasted work in the septum [33].

### CRT-induced recoordination follows a biphasic response

Besides its direct comparison with acute hemodynamic parameters of LV systolic function, the current study extends the findings of previous studies by also demonstrating that the conversion of stretching into shortening follows a biphasic response. Immediately after onset of biventricular pacing, LV coordination improved almost exclusively by a reduction of paradoxical myocardial systolic stretch, whereas longer-term improvements nearly exclusively involved an increase in myocardial systolic shortening (Fig. 2). This biphasic response suggests that, whereas the acute reduction of stretch can be attributed to retiming of ventricular activation and contraction, actual long-term improvements involve secondary mechanisms activated in response to chronic application of CRT.

It is becoming increasingly clear that the chronic response to CRT involves complex molecular, cellular and electrical modifications that are specific to dyssynchronous heart failure and its cure by CRT [15, 16, 25, 34]. Although many of the mechano-feedback pathways remain to be elucidated, abnormal stretch is considered an important mediator for local genetic, cellular and electrical remodelling [25, 35, 36]. Hence, we postulate that restoration of normal contraction patterns may reverse these processes and instigate longer-term improvements. Exemplary in this regard are the findings of Aiba et al. in a canine model of dyssynchronous heart failure. Although immediate restoration of regional strain homogeneity by CRT elicited only minor improvements in LV pump function, considerable reverse molecular remodeling and restoration of regional molecular and electrophysiologic homogeneity ensued [15]*.* We therefore postulate that homogenisation and restoration of normal shortening mechanics play a key role in the mediation of long-term reverse remodelling, that is, in the transition from short to long-term benefit following CRT [25].

Additionally, one of the unique and primary mechanisms by which CRT is believed to convey its longer-term benefits, is through improved myocardial efficiency of ventricular contraction [2, 22, 37]. By definition, segments that shorten against the pressures in systole perform positive work, whereas segments that lengthen (stretch) are subjected to negative (paradoxical) work. Hence, by expressing the fraction of total shortening that is internally dissipated into paradoxical stretch, the internal strain fraction (ISF) conceptually reflects myocardial work inefficiency, or “wasted work” [37, 38]. As such, the observed attenuation of paradoxical systolic stretch at unchanged shortening underlying the immediate ISF-decrease in our study, complies well with the concept that CRT augments cardiac function at unchanged or even diminished energy cost [2]. Conversely, we observed a reduction in end-diastolic volumes paralleled by augmented total shortening at 6 months, a pattern reminiscent of the gradual, true reverse remodelling observed in successful heart failure drug trials.

### Potential clinical implications

Our results show that acute recoordination and functional improvement of LV systolic function are unrelated, and as such question the use of acute hemodynamic improvements as a herald of long-term structural benefit of CRT. Rather, our results point to a prominent role of correction of discoordinated myocardial deformation and in particular the reversal of paradoxical systolic (rebound) stretch as key mechanisms in achieving long-term reverse remodelling and improved LVEF.

Since a stronger reduction of systolic stretching within the septum is associated with a two-fold larger reduction in LVESV, optimizing the extent of recoordination achieved upon initiation of biventricular pacing can be of interest as well [12]. Because CRT can restore discoordination acutely, our results also point to the potential role of discoordination-imaging of as a means of optimization device settings (e.g. electrode selection) directly after CRT implantation, but further research is warranted.

To fulfil its future clinical potential and to allow for integration in prediction models, methods of assessing discoordination will need to be further automatized, quantified and visualized in physiologically meaningful and clinically feasible ways [39, 40]. Promising in this regard are the latest technical advances permitting near-instantaneous, on-scanner implementations of indices such as total and wasted work with encouraging first clinical results.

### Study limitations

Because of non-consecutive enrollment of the patients, the potential influence of selection bias cannot be excluded. Our findings should therefore be confirmed in a larger cohort of unselected patients who underwent CRT implantation. Less preload-dependent hemodynamic measures may be more predicative of a volumetric response than LV dP/dt_max_, SV or LVEF. For example, invasive determination of stroke work was not performed [41]. Also, because no blood pressure data was systematically collected at the time of echocardiography, myocardial wasted work, which incorporates afterload, could not be calculated. The primary goal of the current study was however to explore the mechanisms responsible for CRT response rather than to find the best echocardiographic method to assess those factors. Echocardiography and invasive hemodynamic assessments were not performed simultaneously. All patients were however in a stable clinical condition and echocardiographic examinations were performed within a few days from the implantation procedure. In the majority of cases, baseline and paced LV dP/dt_max_ were measured only once and in non-randomized order. Repeated and randomized measurements, as incorporated in some optimization systems [5], improve the reliability of the measurements and might augment their predictive performance.

## Conclusion

Long-term echocardiographic response after cardiac resynchronisation therapy is likely related to acute recoordination, specifically by attenuation of paradoxical systolic rebound stretch, rather than acute hemodynamic improvement of LV systolic function (Fig. 3). The present findings underscore the relevance of LV recoordination as an *acute* mechanistic pathway for long term reverse remodelling processes after biventricular pacing, and thereby provide physiological insights that further support the ability of discoordination-imaging to predict and asses CRT response [12]. Although consistent, our results are confined to the limitations of a small selected cohort of patients and should be interpreted accordingly.

## Supplementary Information

Below is the link to the electronic supplementary material.

Supplementary file1 (DOCX 1203 KB)

## Data Availability

Data can be made available upon reasonable requests.
