# Supplementary material for: Acute recoordination rather than functional hemodynamic improvement determines reverse remodelling by cardiac resynchronisation therapy
Source: Int J Cardiovasc Imaging. 2021 Feb 5;37(6):1903–11. doi: 10.1007/s10554-021-02174-7 (PMC8255256; doi:10.1007/s10554-021-02174-7)
Supplement: Supplementary file 1 — Supplementary file1 (DOCX 1203 KB) [file 10554_2021_2174_MOESM1_ESM.docx]

**Online supplemental figures**

**Supplemental Fig 1** Calculation of internal stretch fraction

Calculation of internal stretch fraction (ISF) exemplified before, acutely after and six months after CRT. Segmental strain curves of all 18 LV segments (panel A) are first differentiated over time into segmental strain rate curves (panel B). Segmental strain rate curves are then split into a shortening (i.e. negative strain rate) and stretching (i.e. positive strain rate) component. Subsequently, the average strain rate of all LV segments is determined for the shortening and stretching components separately (panel C; dark red and blue lines, respectively). As such, displayed over time, red areas signify the average amount of LV shortening, whereas blue areas signify stretching of the LV. During systole (i.e. between MVC and AVC), overlapping areas (purple) indicate simultaneous systolic shortening and stretching (i.e. ISF). As such, dividing systolic shortening by systolic stretching results in systolic ISF. MVC, mitral valve closure; AVC, aortic valve closure.
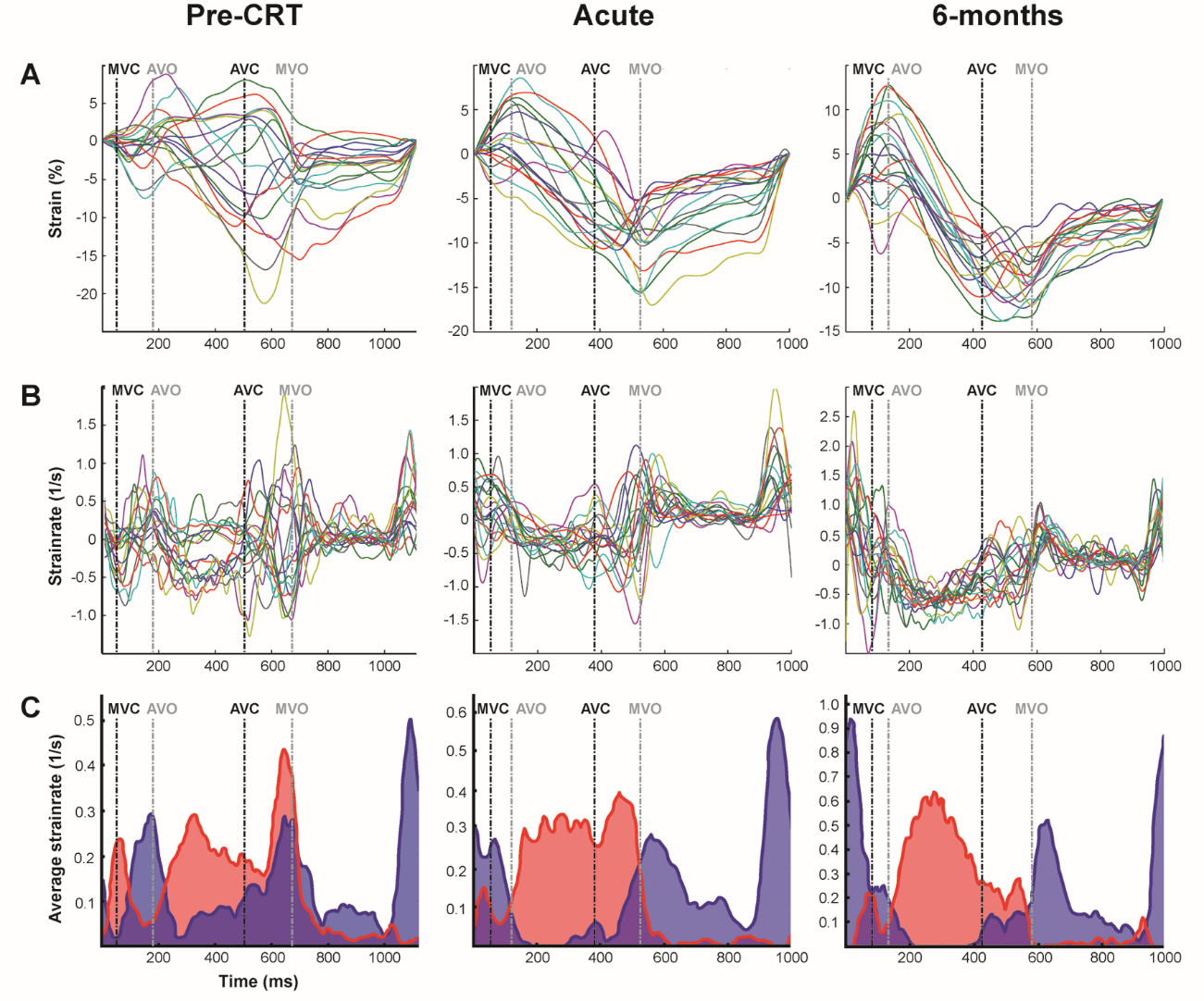


**Supplemental Fig 2** Evolution of dyssynchrony and end-diastolic volume stratified according to response

Mean and standard deviation values of dyssynchrony and left ventricular end-diastolic volume measurements before, directly after and six months after CRT (black, shaded, and white bars, respectively) in responders and non-responders. IVMD, interventricular mechanical delay; 2DS-SD18, standard deviation of time-to-peak strain in 18 segments; LVEDV: left ventricle end-diastolic volume.


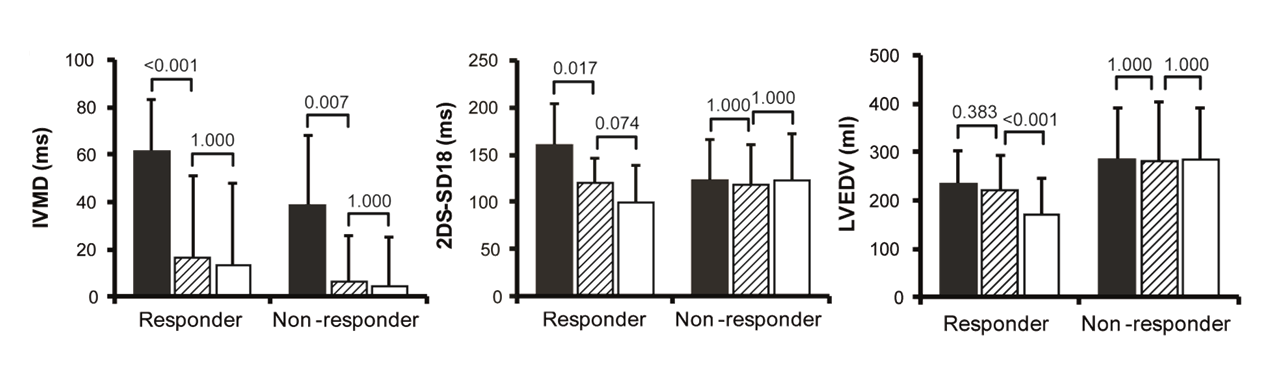


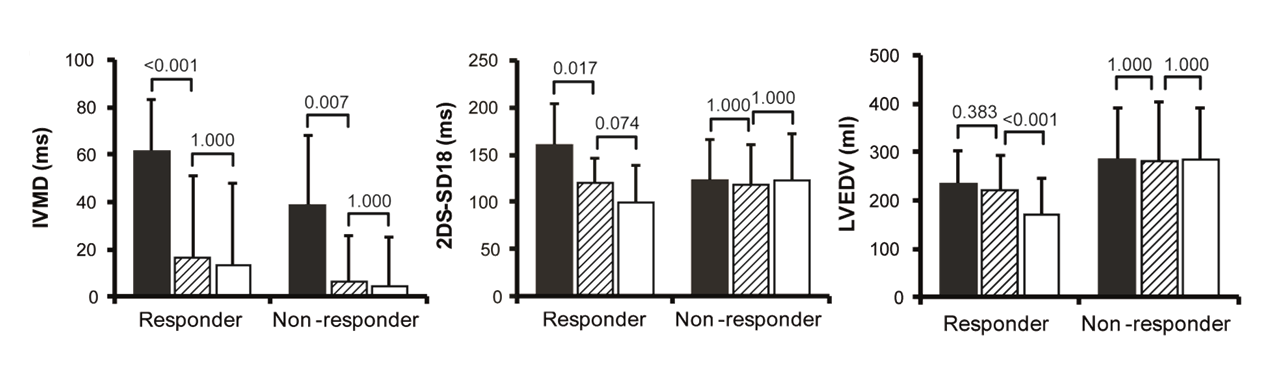
**Online supplemental tables**

Supplemental Table 1: Baseline characteristics of the entire study population and the dP/dt_max_-subgroup

| **Parameter** | | **Responders**  **(n=20)** | **Non-responders**  **(n=15)** | **P-value** | **All Patients (n=35)** | **dP/dt _max_ (n=25)** | **P-value** |
| --- | --- | --- | --- | --- | --- | --- | --- |
| Age (yrs) | | 67±10 | 62±12 | 0.163 | 65±11 | 66±10 | 0.781 |
| Male (%) | | 13 (65) | 8 (53) | 0.511 | 21 (60) | 15 (60) | 1.000 |
| Ischemic aetiology (%) | | 9 (45) | 7 (47) | 1.000 | 16 (46) | 11 (44) | 1.000 |
| QRS (ms) | | 175±22 | 173±33 | 0.789 | 174±27 | 175±28 | 0.906 |
|  | 120 – 150 (%)  > 150 (%) | 3 (15)  17 (85) | 3 (20)  12 (80) |  | 6 (17)  29 (83) |  |  |
| Sinus rhythm (%) | | 19 (95) | 14 (93) | 1.000 | 33 (94) | 23 (92) | 1.000 |
| NYHA class | | 2.9±0.3 | 3.0±0.5 | 0.335 | 2.9±0.4 | 3.1±0.3 | 0.115 |
|  | II  III  IV | 2  18  0 | 2  11  2 |  | 4 29 2 | 0  23  2 |  |
| B-blocker (%) | | 12 (60) | 11 (73) | 0.489 | 23 (66) | 16 (64) | 1.000 |
| ACEi/ARB (%) | | 17 (85) | 15 (100) | 0.244 | 32 (91) | 23 (92) | 1.000 |
| Spironolactone (%) | | 9 (45) | 10 (67) | 0.306 | 19 (54) | 14 (56) | 1.000 |
| Diuretics (%) | | 20 (100) | 15 (100) | 1.000 | 35 (100) | 25 (100) | 1.000 |
| LVEDV (ml) | | 235±68 | 284±101 | 0.096 | 256±86 | 254±88 | 0.914 |
| LVESV (ml) | | 196±58 | 236±99 | 0.143 | 213±79 | 212±82 | 0.971 |
| LVEF (%) | | 18±6 | 19±7 | 0.726 | 19±6 | 18±7 | 0.924 |
| SV (ml) | | 42±14 | 51±16 | 0.088 | 46±15 | 44±14 | 0.670 |
| MRero (mm^2^) | | 6.7±6.9 | 10.7±7.6 | 0.127 | 8.4±7.4 | 9.6±7.7 | 0.252 |
| Heart rate (bpm) | | 68±10 | 69±14 | 0.741 | 69±12 | 71±11 | 0.424 |
| LV lead position (%) | |  |  | 0.129 |  |  | 0.240 |
|  | Post/postlat  Lateral  Anterolateral | 9 (45)  10 (50)  1 (5) | 6 (40)  8 (53)  1 (7) |  | 15 (43)  18 (52)  2 (6) | 11 (44)  12 (48)  2 (8) |  |
| dP/dt_max_ (mmHg/s) | | 713±188 | 618±176 | 0.208 | 668±185 | 668±185 | NA |

NYHA class, New York Heart Association class; ACEi/ARB, angiotensin converting enzyme inhibitor or angiotensin receptor blocker use; LVEDV, left ventricle (LV) end-diastolic volume; LVESV, LV end-systolic volume; SV, stroke volume; LVEF, LV ejection fraction; MRero, mitral regurgitation effective regurgitant orifice; dP/dt_max_, maximum rate of LV pressure rise.

Supplemental Table 2: Overall evolution of discoordination, dyssynchrony, and ventricular function parameters in the entire study population (n=35)

| \| **Parameter** \| **Baseline** \| **Acute** \| **6 Months** \| **P-value** \| \| \| \| --- \| --- \| --- \| --- \| --- \| --- \| --- \| \|  \|  \|  \|  \| **Overall** \| **Baseline vs. Acute** \| **Acute vs. 6 Months** \| \| ISF (%) \| 45±18 \| 27±11 \| 23±12 \| <0.001 \| <0.001 \| 0.022 \| \| Systolic stretch (%) \| 3.86±1.62 \| 2.13±0.48 \| 2.06±0.73 \| <0.001 \| <0.001 \| 1.000 \| \| Systolic shortening (%) \| 8.89±2.35 \| 8.63±2.34 \| 10.24±3.18 \| <0.001 \| 1.000 \| 0.002 \| \| SRSlv (%) \| 2.27±1.33 \| 0.74±0.50 \| 0.71±0.43 \| <0.001 \| <0.001 \| 1.000 \| \| 2DS-SD18 \| 144±45 \| 121±33 \| 111±44 \| 0.004 \| 0.066 \| 0.468 \| \| IVMD \| 52±27 \| 13±29 \| 10±28 \| <0.001 \| <0.001 \| 1.000 \| \| LVEDV (ml) \| 256±86 \| 249±97 \| 219±104 \| <0.001 \| 0.585 \| <0.001 \| \| LVESV (ml) \| 211±80 \| 195±87 \| 167±96 \| <0.001 \| 0.005 \| 0.001 \| \| LVEF (%) \| 19±7 \| 23±8 \| 27±10 \| <0.001 \| <0.001 \| 0.007 \| \| SV (ml) \| 46±15 \| 54±20 \| 52±16 \| 0.003 \| 0.025 \| 1.000 \| \| MRero (mm^2^) \| 8.4±7.4 \| 5.9±5.5 \| 5.9±6.4 \| 0.010 \| 0.006 \| 0.334 \| \| TAPSE (mm) \| 15.4±5.6 \| 16.3±4.0 \| 17.1±4.9 \| 0.043 \| 0.144 \| 0.366 \| \| Heart rate (bpm) \| 69±12 \| 69±11 \| 68±9 \| 0.675 \| 1.000 \| 0.782 \| \| dP/dtmax (mmHg/s) \| 668±185 \| 817±198 \| - \| - \| <0.001 \| - \| |
| --- | --- | --- | --- | --- | --- | --- | --- | --- | --- | --- | --- | --- | --- | --- | --- | --- | --- | --- | --- | --- | --- | --- | --- | --- | --- | --- | --- | --- | --- | --- | --- | --- | --- | --- | --- | --- | --- | --- | --- | --- | --- | --- | --- | --- | --- | --- | --- | --- | --- | --- | --- | --- | --- | --- | --- | --- | --- | --- | --- | --- | --- | --- | --- | --- | --- | --- | --- | --- | --- | --- | --- | --- | --- | --- | --- | --- | --- | --- | --- | --- | --- | --- | --- | --- | --- | --- | --- | --- | --- | --- | --- | --- | --- | --- | --- | --- | --- | --- | --- | --- | --- | --- | --- | --- | --- | --- | --- | --- | --- | --- | --- | --- |

Abbreviations: ISF, internal stretch fraction; SRSlv, left ventricular systolic rebound stretch; 2DS-SD18, standard deviation of time to peak strain; IVMD, interventricular mechanical delay; LVEDV, left ventricular (LV) end-diastolic volume; LVESV, LV end-systolic volume; LVEF, LV ejection fraction; SV, stroke volume; MRero, mitral regurgitation effective regurgitant orifice; TAPSE, tricuspid annular plane systolic excursion; dP/dt_max_, maximum rate of LV pressure rise.Supplemental Table 3: Relation of acute recoordination & resynchronisation parameters with those representing acute hemodynamic improvement (n=25)

| **Parameter** | | | **Acute ∆dP/dt_max_ (%)** | | **Acute ∆SV**  **(%)** | | **Acute ∆LVEF**  **(%-point)** | |
| --- | --- | --- | --- | --- | --- | --- | --- | --- |
|  | | | **R** | **p-value** | **R** | **p-value** | **R** | **p-value** |
| Acute recoordination | | |  |  |  |  |  |  |
|  | | ∆ISF (%-point) | 0.201 | 0.334 | 0.005 | 0.980 | 0.077 | 0.720 |
|  | | ∆SRSlv (%-point) | -0.050 | 0.814 | -0.079 | 0.720 | -0.186 | 0.385 |
| Acute resynchronisation | | |  |  |  |  |  |  |
|  | ∆IVMD (ms) | | 0.381 | 0.080 | 0.309 | 0.186 | 0.346 | 0.125 |
|  | ∆2D-SD18 (ms) | | 0.302 | 0.143 | 0.089 | 0.685 | 0.317 | 0.131 |

For a uniform representation, all changes ∆ express a physiologic improvement, i.e. decrements for discoordination & dyssynchrony parameters, and increments for function parameters such that relations are positive if both parameters improve. Other abbreviations: see Table 1 in the main document.
